# Linking Salivary Calbindin-D28k to Oral Health Outcomes in Chronic Kidney Disease

**DOI:** 10.1055/s-0045-1809976

**Published:** 2025-07-18

**Authors:** Mohamed Abdullah Jaber, Alexander Maniangat Luke, Mohamed Saleh Hamad Ingafou, Nireeksha Nireeksha

**Affiliations:** 1 Department of Clinical Science, College of Dentistry, Ajman University, Ajman, United Arab Emirates; 2 Centre of Medical and Bio-Allied Health, Science Research, Ajman University, Al- Jurf, Ajman, United Arab Emirates; 3 Department of Conservative Dentistry and Endodontics, AB Shetty Memorial Institute of Dental Sciences (ABSMIDS), Nitte (Deemed to be University), Deralakatte, Mangalore, Karnataka, India

**Keywords:** calbindin-D28K, renal dialysis, oral health, chronic kidney disease (CKD)

## Abstract

**Objective:**

Chronic kidney disease (CKD) is a universal health concern, with CBD- 28k (calbindin-D28k) being 1 alpha,25-dihydroxyvitamin D3-dependent calcium-binding protein, playing a role in kidney function. Renal dialysis may alter calbindin-D28k levels, impacting oral health and periodontal health. Understanding these impacts is crucial for managing CKD. This study aimed to explore the potential of salivary calbindin-D28k as a predictor of oral health in subjects with CKD.

**Materials and Methods:**

This case–control study involved 200 individuals aged 24 to 77 years, out of which 100 were undergoing CKD treatment. Biochemical parameters were recorded, and unstimulated saliva samples were collected. Decayed, Missing and Filled Teeth and periodontal scores were noted after clinical examination. Salivary calbindin-D28k level was estimated by immunosorbent assay (enzyme-linked immunosorbent assay). A two-tailed test of *p* -value of < 0.05 was used to determine statistics.

**Results:**

Demographic parameters showed a significant age and gender difference between the groups. Significant variation in the level of hematological and biochemical parameters between the groups was observed. Renal function test and liver function test levels varied significantly in CKD patients. Notable difference in the level of salivary calbindin-D28k levels and periodontal scores revealed poor oral health. The best cutoff value for calbindin-D28k was obtained from the receiver operating characteristic curve indicating a risk of poor oral health in CKD subjects. Additionally, salivary calbindin-D28k levels and oral health showed significant association and correlation.

**Conclusion:**

The current research findings suggest that monitoring salivary calbindin-D28k levels could serve as an important predictor of oral health in CKD subjects highlighting the importance of complete oral health management in subjects with CKD.

## Background

Chronic kidney disease, or CKD, is a serious worldwide health issue. Approximately 8 to 10% of people worldwide suffer from mild to moderate renal impairments, and the number of instances of CKD is rapidly increasing. 1 Like other systemic diseases, CKD is linked to oral health issues that are brought on by the condition or its management. Their prognosis and general oral health may worsen if these lesions are not treated. 2 Calcium ions (Ca2 ^+^ ) are firmly bound by soluble intracellular proteins called calbindins, which are vitamin D-dependent. One of the main calbindins found in many mammalian organs, including the kidney, is calbindin-D28k protein (calbindin-D28k-1 alpha,25-dihydroxyvitamin D3-dependent calcium-binding protein). It plays a crucial role in calcium transport and intracellular calcium level regulation, both of which are essential for renal calcium absorption and healthy kidney function, and most importantly it absorbs calcium in micromolar range. Vitamin D3 binds to its receptor in the regulatory region of calbin-D28k and stimulates synthesis of calbindin, that is, transcription of calbindin-D28k. Therefore, biologically active vitamin D (1,25-dihydroxy vitamin D) is important for the availability of levels of calbindin-D28k. In patients with renal dialysis, oral health poses a potential predictor of health outcomes due to the effect of variation in vitamin D3 levels and, therefore, calbindin-D28k. 3 4 Compared to the general population, adults with renal disease have been found to have a wide range of oral complications, such as caries, periodontitis, altered salivary composition and pH, reduced salivary flow, enhanced dental calculus configuration, elevated salivary buffering capacity, and decreased levels of calcium. Additionally, poor oral hygiene has been linked to higher mortality rates. 5 CKD impairs vitamin D, mineral metabolism with reduced calcium reabsorption, and decreased antimicrobial property of antimicrobial peptides. Calbindin-D28k is believed to perform a functional role in controlling the reabsorption of calcium in the distal nephron and vitamin D3 plays a potential role in distal convoluted tubule (DCT). 6 7 Tooth needs calcium for its maturation, these calcium ions are transported by cytosolic calbindin-D28k, a protein that is found in the kidney's DCT, which plays an important role in active calcium transport and antimicrobial property. Renal dialysis may alter CBD-28k levels, impacting oral health and periodontal health. Although marked variations in the level of calbindin-D28k expression have been observed in other diseases, recent study suggests a possible association between calbindin-D28k (CBD-28k), a calcium-binding protein implicated in calcium homeostasis, and dental health in individuals receiving renal dialysis. Nonetheless, concrete data demonstrating a causal connection between calbindin-D28k levels and oral health outcomes in this cohort is absent. Although research has noted modifications in salivary content and a heightened incidence of oral problems, including caries and periodontal disease, in dialysis patients, the precise function of calbindin-D28k in these alterations has yet to be elucidated. In light of these information gaps, this work seeks to examine the correlation between calbindin-D28k expression and oral health measures in persons undergoing renal dialysis, to further understanding of the underlying processes and potential therapeutic ramifications. 8 9

Their role in CKD individuals with poor oral health is less studied. Hence, the present study aims to investigate the potential of salivary calbindin-D28k as a predictor of oral health in subjects with CKD, offering valuable insights into the interplay between renal function and oral health.

## Material and Methods

### Study Design

This case–control study was carried out at Justice K.S. Hegde Charitable Hospital, Nitte Deemed to be University, AB Shetty Memorial Institute of Dental Sciences (ABSMIDS) and Central Research Laboratory (K.S. Hegde Medical Academy) Deralakatte, Mangalore, Karnataka, India. The study was approved by the Nitte (Deemed to be University) Ethics Committee (Approval number: NU/CEC/2023/0215), dated: August 20, 2023.

### Subjects

A total of 100 subjects diagnosed with CKD and 100 age-matched individuals between 24 and 77 years were included after obtaining informed consent.

The sample size was calculated using:



where expected odds ratio (OR) = 2.0, *P*_0_ = 0.30, *α* = 0.05 (95% confidence level), power = 0.90, and case–control ratio = 1:1.

The case's inclusion criteria comprised adult CKD subjects in the predialysis state with a Decayed, Missing, and Filled Teeth (DMFT) (dental caries prevalence) of > 3. While for the control, age-matched healthy individuals without CKD with a DMFT < 0 visiting the outpatient clinic for routine examinations were included. The study excluded subjects with other systemic diseases, pregnancy, and any other malignant diseases.

### Baseline Questionnaire

Basic detailed information regarding the patient's details was recorded in a predesigned pro forma and medical records were used to record biochemical parameters.

### Clinical Examination

The number of caries-related decaying (D), filled (F), and missing (M) teeth was calculated based on the clinical examination results and the DMFT score of the samples. Data was gathered by questions by means of direct examination and observation of the subjects' teeth using a Medisporex catheter and mirror number 4. Dental mouth mirror and probes were used to examine dental caries, and following each patient's examination, the periodontal score was calculated and the results were documented in the pro forma.

### Sample Collection

Individuals were restrained from eating 2 hours prior sample collection, mouth was rinsed with normal water, and samples were collected in between 9 and 10 a.m. to maintain the time duration and uniformity among the individuals. A sample of unstimulated saliva was collected on the mouth's floor. A Tarson saliva collection tube was used to collect 5 mL. The patient's saliva was collected, centrifuged, and the supernatant was kept for further examination at –20°C.

### Biochemical Estimation

Saliva calbindin-D28k was estimated by enzyme-linked immunosorbent assay (ELISA; using a commercially available kit [Cat. No. LAB3789], Labrecon, India) and measured using an ELISA reader (Spark Tecan).

### Statistical Analysis

The statistical software SPSS (IBM, version 22.0, SPSS Inc, Chicago, Illinois, United States) was used to analyze the data. Qualitative data were represented as numbers and percentages. Quantitative data of both cases and controls were reported as mean ± standard deviation for normally distributed data by applying an unpaired independent *t* -test and for skewed data, Mann–Whitney *U* test was applied for the median-interquartile range. Pearson's and Spearman's correlation coefficients were used for correlation studies. The association between salivary calbindin-D28k levels and oral health was determined using the chi-square test and by calculating the OR at a 95% confidence interval. A *p* -value of less than 0.05 was considered statistically significant in relation to the groups.

## Results

### To Study the Baseline Characteristics With and Without Kidney Dialysis

This case–control study had 200 subjects, out of which 100 subjects were diagnosed with CKD in the predialysis stage with a poor oral health, DMFT of > 3. Whereas subjects without CKD and good oral health with a DMFT of 0 were considered controls. Table 1 provides baseline characteristics of the study population, showing a considerable difference in age between the two groups. While comparing the gender between the groups, the percentage of male population was higher in case when compared to control and it was statistically significant. The percentage of periodontal score 3 4 was significantly high in case subjects compared to control.

**Table 1:** Baseline characteristics of the study population

Parameters	Case ( *n* = 100)	Control ( *n* = 100)	*p* -Value
Age	52.21 ± 11.68	30.26 ± 5.59	** 0.000 a**
DMFT score	2.01 ± 0.62	0.36 ± 0.48	0.272
Gender (%) Male Female	58.33 41.66	26.66 73.33	** 0.000 a**
Periodontal scoring (%) 0–2 3–4	40 60	96.66 3.33	** 0.000 a**

Abbreviations: DMFT, Decayed, Missing and Filled Teeth; SD, standard deviation.

Note: Age and DMFT scoring is represented as mean ± SD. Gender and periodontal scoring is expressed in percentage (%). *p* -Value was calculated using the Student's *t* -test for parametric variables.

a *p* < 0.0001. The statistical significance of case–control differences was tested for categorical variables using a chi-square test.

### Hematological and Biochemical Characteristics in Subjects with Chronic Kidney Disease and Control

While comparing the hematological and biochemical characteristics between the group, the level of hemoglobin, bicarbonate (mmol/L), sodium (mmol/L), and calcium (mg/dL) was significantly less in subjects with CKD and poor oral health. Whereas phosphate-buffered saline, random blood sugar (RBS), phosphorous (mg/dL), and potassium (mmol/L) levels were significantly high in case subjects when compared to control. Hematological and biochemical characteristics of the study population are given in Table 2 .

**Table 2:** Hematological and biochemical characteristics of the study population

Biochemical parameters	Case ( *n* = 100)	Control ( *n* = 100)	*p* -Value
Hemoglobin (g/dL)	10.05 (8.70–10.65)	11.90 (11.70–12.57)	** 0.002 b**
PBS (mg/dL)	152.48 (142–160)	100 (97–102)	** 0.032 a**
RBS (mg/dL)	129 (114–146)	100 (97.0–102)	** 0.000 b**
Albumin (g/dL)	3.90 (3.60–4.20)	4.20 (4.00–4.45)	0.086
Bicarbonate (mmol/L)	9.0 (6.1–12.0)	22.0 (21.00–23.00)	** 0.000 b**
Phosphorous (mg/dL)	6.95 (4.00–6.37)	3.00 (2.80–3.1)	** 0.002 b**
Sodium (mmol/L)	128.00 (125.00–143.00)	138.5 (137.00–139.00)	** 0.000 b**
Potassium (mmol/L)	5.20 (4.50–5.47)	4.00 (3.90–4.00)	** 0.000 b**
Calcium (mg/dL)	6.05 (5.50–8.50)	9.00 (9.00–9.30)	** 0.012 b**

Abbreviations: PBS, phosphate-buffered saline; RBS, random blood sugar.

Note: Biochemical parameters are represented as median values (interquartile range). *p* -Value was calculated using the Kruskal–Wallis test.

a *p* < 0.001 was considered statistical significant.

b *p* < 0.0001 was considered statistical significant.

### Estimate of Salivary Calbindin-D28k Levels in Subjects With and Without Kidney Dialysis

**Fig. 1:**
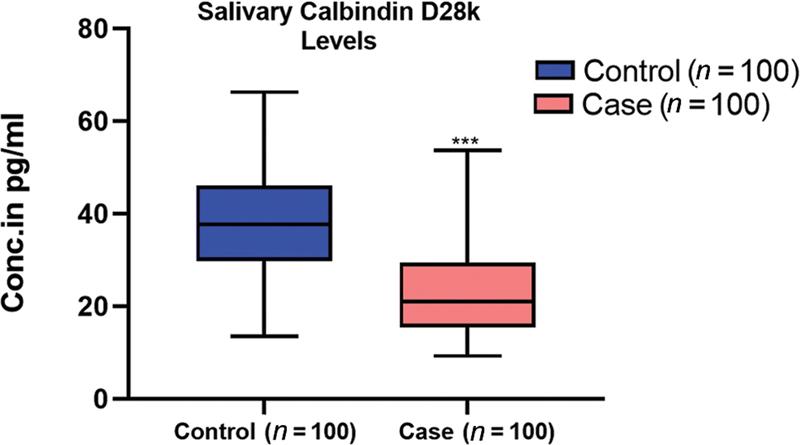
Salivary levels of calbindin-D28k (pg/mL) between the chronic kidney disease case and control subjects; Data are shown as median (interquartile range); *p* -value was calculated using the Mann–Whitney *U* test. *** *p* < 0.0001, indicated as significant.

Further, in our major topic of interest, we estimated salivary calbindin-D28k levels in subjects with and without CKD and we observed a significant reduction in the level of salivary calbindin-D28k levels in subjects with CKD and poor oral health. The result obtained is shown in Fig. 1 .

### Evaluation of the Association between the Intracellular Protein Calbindin-D28k in Patients with Renal Dialysis and Their Oral Health

**Fig. 2:**
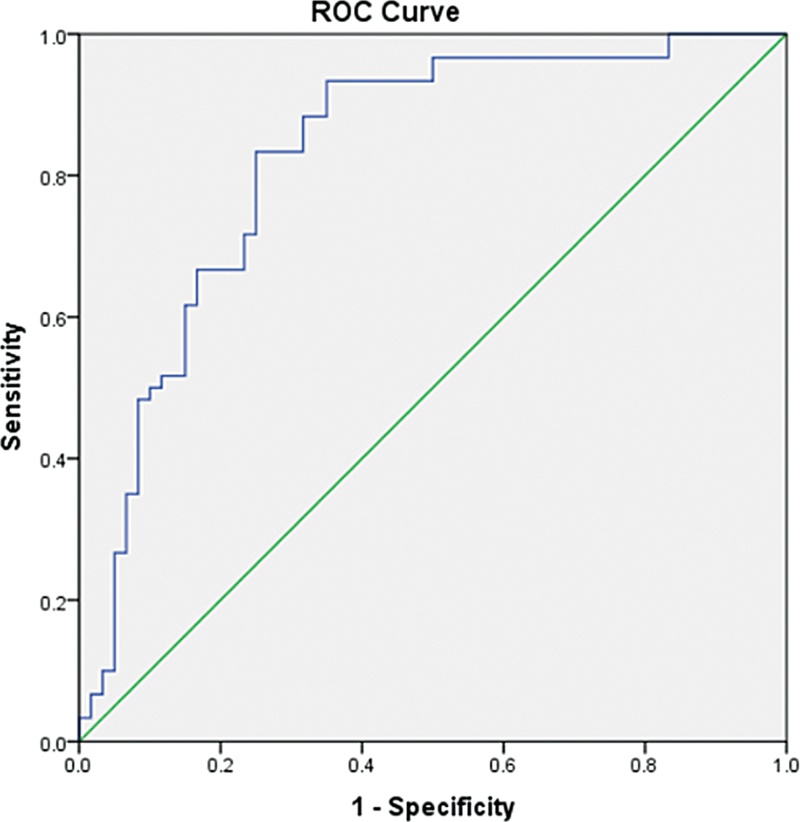
The area under the receiver operating characteristic (ROC) curve for the levels calbindin-D28k was 0.828, the best cutoff value was 29.04 pg/mL.

Further, to find the association of calbindin-D28k in subjects with CKD and their oral health we obtained the best cutoff value for salivary calbindin-D28k using the receiver operating characteristic (ROC) curve, that is, 29.04 pg/mL, with the area under the curve of 0.828, and the results obtained were significant. The results are depicted in Fig. 2 and Table 3 .

**Table 3:** To evaluate the association of intracellular protein calbindin-D28k in patients with renal dialysis and their oral health

Area under the curve	Sensitivity	1-specificity	*p* -Value
0.828	0.767	0.250	0.000 a

Note: *p* -Value was calculated at 95% confidence.

a *p* < 0.0001 was considered as statistically significant. The area under the receiver operating characteristic (ROC) curve for the levels of calbindin-D28k was 0.828, the best cutoff value was 29.04 pg/mL.

### Risk Estimates of Oral Health Based on the Levels of Calbindin-D28k in Patients with Chronic Kidney Disease

After obtaining the best cutoff value for calbindin-D28k, we estimated the risk of oral health based on the levels of calbindin-D28k in subjects with CKD. Here, we understood that the lower level of salivary calbindin-D28k in subjects with CKD were associated with the higher risk of developing poor oral health. This was confirmed with the higher periodontal score obtained in case subjects with CKD ( Table 4 ).

**Table 4:** Risk estimates of oral health based on the levels of calbindin-D28k in patients with renal dialysis

Parameter		Control ( *n* = 100)	Case ( *n* = 100)	Odds ratio (95% CI)	*p* -Value
Salivary calbindin-D28k	Low	20	75	0.26 (0.15–0.45)	** 0.000 a**
	High	80	15	3.20 (2.02–5.05)	
Periodontal score	Low	83.33	41.66	0.50 (0.363–0.689)	**0.** 000 **a**
	High	16.66	58.33	3.50 (1.91–6.40)	

Abbreviations: CI, confidence interval; ROC, receiver operating characteristic.

Note: The area under the ROC curve for the levels of calbindin-D28k was 0.828, the best cutoff value was 29.04 pg/mL.

a *p* < 0.0001were considered to indicate statistical significance.

The obtained cutoff value of periodontal scoring is 3 (greater than 3 is considered as worst and lesser than 3 is considered as good). Which was further divided into lower and higher value groups, *p* -value derived from the chi-square test for each parameter.

### Analyzing Biochemical Parameters in Patients with Chronic Kidney Disease

Further, we analyzed the biochemical parameters in patients with CKD ( Table 5 ). Here, we observed significant increase in the level of alkaline phosphatase (ALP) (U/L), serum glutamic oxaloacetic transaminase (SGOT) (U/L), and serum glutamic pyruvic transaminase (SGPT) (U/L) in liver function test (LFT) of case subjects when compared to control. Whereas in renal function test (RFT), we observed that the urea (mg/dL), creatinine (mg/dL), and uric acid (mg/dL) were significantly higher in case subjects.

**Table 5:** Biochemical parameters in patients with chronic kidney disease

LFT	Case ( *n* = 100)	Control ( *n* = 100)	*p* -Value
Alkaline phosphatase (U/L)	148.00 (107–267.25)	51.00 (50.00–53.00)	**0.000** a
SGOT (U/L)	21.00 (15.45–26.90)	17.00 (15.00–19.00)	**0.000** a
SGPT (U/L)	16.50 (9.00–20.00)	14.00 (14.00–17.00)	**0.000** a
RFT			
Urea (mg/dL)	103.00 (75.00–110.75)	19.00 (18.00–21.00)	**0.000** a
Creatinine (mg/dL)	10.10 (8.40–11.70)	0.70 (0.70–0.70)	**0.000** a
Uric acid (mg/dL)	5.70 (3.70–7.1)	3.50 (3.20–3.80)	**0.000** a

Abbreviations: LFT, liver function test; RFT, renal function test; SGOT, serum glutamic oxaloacetic transaminase; SGPT, serum glutamic pyruvic transaminase.

Note: Biochemical parameters are represented as median values (interquartile range). *p* -Value was calculated using the Kruskal–Wallis test.

a *p* < 0.0001 was considered statistically significant.

### Association of Intracellular Protein Calbindin-D28k in Patients with Renal Dialysis and Their Oral Health

Further, while associating calbindin-D28k in patients with renal dialysis with their oral health, periodontal scoring showed significant association with salivary calbindin-D28k ( Table 6A ). The association of salivary calbindin-D28k was continued with RFT and LFT, and a significant result was observed between RFT and salivary calbindin-D28k levels ( Table 6C ); this table indicates that individuals with lower level of calbindin-D28k were significantly associated with RFT. However, the association between LFT and salivary calbindin-D28k did not show significant result. The area under the ROC curve for the levels calbindin-D28k was 0.828, the best cutoff value was 29.04 pg/mL. The obtained cutoff value of periodontal scoring is 3 (greater than 3 is considered as worst and lesser than 3 is considered as good). Which was further divided into lower and higher value groups, *p* -value derived from the chi-square test for each parameter ( Table 6B ).

**Table 6:** Association of intracellular protein calbindin-D28k in patients with periodontal scoring in individuals with chronic kidney disease

(A)			
Parameter	Periodontal score		*p* -Value
	High (58.33)	Low (41.66)	
Salivary calbindin-D28k	21.11 (15.40–29.41)		** 0.009 b**
**(B)**			
LFT	** Salivary calbindin-D28k**		***p*** **-Value**
	**High** **(15)**	**Low** **(45)**	
Alkaline phosphatase (U/L)	148.00 (107–267.25)		0.097
SGOT (U/L)	21.00 (15.45–26.90)		0.152
SGPT (U/L)	16.50 (9.00–20.00)		0.201
**(C)**			
RFT	**Salivary** **calbindin-D28k**		***p*** **-Value**
	**High** **(15)**	**Low** **(45)**	
Urea (mg/dL)	103.00 (75.00–110.75)		0.096
Creatinine (mg/dL)	10.10 (8.40–11.70)		** 0.05 a**
Uric acid (mg/dL)	5.70 (3.70–7.1)		** 0.05 a**

Abbreviations: LFT, liver function test; RFT, renal function test; SGOT, serum glutamic oxaloacetic transaminase; SGPT, serum glutamic pyruvic transaminase.

Note: The parameters are represented as median values (interquartile range).

a *p* ≤ 0.05 considered to indicate statistical significance.

b *p* ≤ 0.001 was considered to indicate statistical significance.

### Correlation between Intracellular Protein Calbindin-D28k in Patients with Renal Dialysis and Their Oral Health

Further, we performed a correlation analysis between the intracellular protein calbindin-D28k in patients with renal dialysis and their oral health. Here, we observed significant positive correlation between intracellular protein calbindin-D28k and biochemical parameters such as sodium and RBS, whereas significant negative correlation was observed between calcium and salivary calbindin-D28k levels ( Table 7A ). Further, we continued correlating calbindin-D28k with periodontal scoring and liver health and renal health and we obtained significant negative correlation between salivary calbindin-D28k levels and periodontal scoring in cases with CKD and poor oral health ( Table 7B ).

**Table 7:** Correlation between intracellular protein calbindin-D28k in patients with chronic kidney disease and their oral health

(A)										
**Variables**		**HB**	**Sodium**	**Potassium**	Albumin	Phosphorous	Calcium	Bicarbonate	PBS	RBS
Salivary calbindin-D28k	*R*	0.023	0.296	0.014	0.134	–0.036	–0.326	–0.066	–0.053	0.287
	Sig	0.864	** 0.022 a**	0.915	0.308	0.787	** 0.011 a**	0.614	0.686	** 0.026 a**
**(B)**										
**Variables**				**Oral health**	**Liver health (LFT)**			**Renal health (RFT)**		
				**Periodontal** **scoring**	**ALP**	**SGOT**	**SGPT**	**Urea**	**Creatinine**	**Uric acid**
Salivary calbindin-D28k		*r*		–0.265	0.089	–0.139	0.093	–0.063	0.077	0.113
		Sig		** 0.04 a**	0.501	0.291	0.478	0.630	0.557	0.390

Abbreviations: ALP, alkaline phosphatase; HB, ; hemoglobin; LFT, liver function test; PBS, phosphate-buffered saline; RBS, random blood sugar RFT, renal function test; SGOT, serum glutamic oxaloacetic transaminase; SGPT, serum glutamic pyruvic transaminase.

Note: Spearman's correlation was done for all nonparametric variables.

a *p* ≤ 0.05 is considered as statistically significant (two-tailed).

## Discussion

Individuals with CKD frequently have variations in their oral cavities, such as periodontitis, and different signs of oral health problems can lead to morbidity and death. 10 11 12 13 In the current study, the age of subjects on kidney dialysis was between 24 and 77 years, and the mean age was 52.21 years, while the male population was higher, and both age and gender significantly differed between the two groups. Clinical assessment revealed that the population under study had a very high DMFT index score on average, which was not significant, while the periodontal score was significantly higher in case subjects. The data indicates that CKD is most prevalent among patients in their 50s, Additionally, the study suggests a slight male predilection for CKD, aligning with previous research. 14 15 16 High DMFT score in CKD subjects corroborated with previous research. 17 18 CKD has been empirically linked to dental deterioration, culminating in tooth loss. Additionally, CKD adversely affects the oral functioning. Numerous studies have documented a heightened prevalence of oral pathologies among patients on dialysis with few to multiple oral complications 19 leading to have implications for dental treatment. 20 The DMFT index has been the most significant indicator for evaluating the state of oral and dental health for more than 70 years, both internationally and locally, while periodontal scoring system used to evaluate interproximal gingival health. Patients with CKD may have higher rates of periodontitis and dental caries, which increases the risk of tooth loss and difficulty chewing due to insufficient occlusive surfaces or prosthetic constraints. 21 The pathogenesis of deteriorating oral health can be attributed to the increased intracellular calcium ions. Calbindin-D28K is located in DCT and coexists with active vitamin D. The impaired function of calbindin-D28k prevents absorption and regulation of calcium levels. Free calcium ions perform various biological roles or functions in cancer cell growth inhibitors, tumor invasiveness, and drug resistance. Intracellular calcium overload prevents membrane localization and may result in apoptosis of cells. Therefore, may directly affect the periodontal tissues and provide ideal environment for growth of bacteria to cause tooth surface demineralization. 22 Prior research has found that the CKD patients in the study had noticeably higher plaque scores when compared to healthy controls. 23 24 25 The use of antidiuretic medications, which may reduce salivary flow and lubricate the oral cavity less effectively, may be connected to the association between poor oral hygiene and CKD. This could lead to an increased risk of plaque accumulation. Additionally, these patients' oral hygiene may have gotten worse due to inadequate oral health care practices. 14

When the hematological and biochemical characteristics of case subjects were compared with control, we observed a decline in hemoglobin, bicarbonate, sodium, and calcium levels in CKD subjects with poor oral health. More precisely, anomalies in the metabolism of phosphate and calcium can affect tooth mineralization and periodontal health, suggesting a potential link with calcium-regulating proteins such calbindin-D28k. Pallor of the mucosa, primarily from anemia (lower erythropoietin synthesis), is the most frequent oral finding in dialyzed patients with reference to the mucosal and glandular involvement. 26 27 Renal anemia and changes in platelet aggregation maintain these patients' propensity to bleed. 26 28 Furthermore, hemodialysis increases the risk of oral mucosal bleeding, petechiae, and ecchymoses. 29 30 It is imperative to evaluate the accessibility of dental health services for individuals suffering from CKD. This emphasizes the need for a customized oral health program that is both preventive and therapeutic, as well as ongoing follow-up to help these subjects understand the importance of oral health given their systemic illness. 31

Further, to understand the role of salivary calbindin-D28k in oral health, a significant decrease in salivary calbindin-D28k levels in subjects with CKD and poor oral health was observed. Calbindin expression in ameloblasts has also been reported by numerous researchers, and CB28k has been detected in a variety of mammalian tissues. Moreover, CB28k has been detected in cells of the epithelial rests of Malassez, odontoblasts, and fibroblasts found in periodontal ligaments, as well as in places devoid of enamel. 6 Calbindin-D28k is known for its role in facilitating calcium absorption and maintaining calcium balance within cells. The significant reduction in salivary calbindin-D28k levels in CKD patients with poor oral health could have several implications. First, it may indicate a systemic disruption of calcium metabolism, which is commonly seen in CKD due to impaired renal function. The kidneys play a crucial role in regulating calcium and phosphate levels, and dysfunction in these organs can lead to imbalances that affect various tissues, including the oral cavity. In the context of oral health, decreased salivary calbindin-D28k levels could contribute to weakened dental structures and increased susceptibility to caries and other oral diseases. Calcium is vital for maintaining the integrity of enamel and dentin, and reduced levels of calbindin-D28k might result in insufficient calcium availability in saliva, thereby compromising the remineralization process of teeth. This could partly explain the high prevalence of advanced carious lesions, as indicated by the elevated PUFA (Pulpal Involvement, Ulceration, Fistula, Abscess) index in CKD patients. Moreover, the reduction in salivary calbindin-D28k levels might also be associated with a diminished ability to buffer acids in the oral environment, leading to an increased risk of dental erosion and caries formation.

In this study, there was a significant association between the intracellular protein calbindin-D28k and periodontal scoring in chronic renal dialysis patients with oral health problems with ROC of 0.828. The lower level of salivary calbindin-D28k in subjects with CKD was associated with the higher risk of developing poor oral health, confirmed with periodontal scoring. Ameloblasts, odontoblasts, and cementoblasts are highly differentiated cells that generate highly mineralized tissues that make up mammalian teeth. One important function of Ca2+ deposition in these cells is to facilitate their formation. Ameloblasts have been demonstrated to contain calbindin-D28k in a number of studies. 6 32 33 Patients on dialysis may have variations in calbindin-D28k expression, which could affect the teeth's mineralization process and increase their susceptibility to dental caries and other mineralization issues. Calbindin-D28k may have an effect on periodontal health via controlling calcium levels.

The association between salivary calbindin-D28k and RFT was significant, but it did not show significance with LFT. Because calbindin-D28k is expressed and regulated differently in different tissues, there is no discernible correlation between calbindin-D28k (Calb1) and LFTs. In order to support intracellular calcium transport and buffering, this calcium-binding protein is primarily expressed in calcium-transporting organs such the kidney, gut, and certain brain areas. On the other hand, it has little to no expression in the liver, suggesting that it plays a little part in the handling or metabolism of calcium in the liver. Additionally, the active form of vitamin D (1,25-dihydroxyvitamin D3) regulates calbindin-D28k expression in a tissue-specific way. Although vitamin D dramatically increases calbindin-D28k in the kidney and gut, the liver does not exhibit this regulating effect, indicating that calbindin-D28k has a restricted function in the liver. The analysis of the association between salivary calbindin-D28k levels and RFTs versus LFTs in patients with CKD provides important insights into the specific roles that different organ systems play in regulating this calcium binding. 34 The significant association observed between salivary calbindin-D28k levels and RFTs underscores the vital role of kidney function in the regulation and expression of calbindin-D28k. Calbindin-D28k is a calcium-binding protein that plays a key responsibility in calcium homeostasis, and the kidneys are central to maintaining calcium and phosphate balance in the body. In CKD, where renal function is impaired, the dysregulation of calcium metabolism can lead to alterations in the levels of calcium-binding proteins like calbindin-D28k. The significant correlation suggests that as kidney function declines, there may be compensatory or pathological changes in the production or secretion of calbindin-D28k, which is reflected in its salivary levels. This association is particularly relevant given the role of calbindin-D28k in various physiological processes, including its potential impact on bone health and dental structures. In CKD patients, impaired kidney function often leads to disturbances in calcium and phosphate metabolism, which can manifest in various systemic and oral health issues, including periodontal disease and other oral pathologies. The significant relationship between calbindin-D28k and RFT highlights the potential of this protein as a biomarker for assessing the extent of renal dysfunction and its systemic repercussions, including its effects on oral health. On the other hand, the lack of a significant association between salivary calbindin-D28k levels and LFTs suggests that liver function does not play a major role in regulating this protein, at least not in the context of CKD. While the liver is involved in various metabolic processes and the synthesis of many proteins, the regulation of calbindin-D28k appears to be more closely tied to renal function. This finding aligns with the understanding that calbindin-D28k is primarily involved in calcium homeostasis, a process in which the kidneys play a more direct and pivotal role compared to the liver. The absence of a significant association with LFT indicates that hepatic function, despite its broad role in metabolism, may not directly influence the levels of calbindin-D28k in saliva. 35

The biochemical tests such as LFT (ALP, SGOT, and SGPT) and RFT (urea, creatinine, and uric acid) levels were significantly higher in CKD subjects than control. Calcium reabsorption is decreased in CKD, which also affects vitamin D and mineral metabolism. CKD or its therapy can cause a number of variations in the oral cavity, even though there are no specific symptoms of the disease. 36 The analysis of biochemical parameters in subjects with CKD provides valuable insights into the systemic alterations associated with CKD and their potential impact on oral health, particularly periodontal status. The association between high serum urea and creatinine levels and poor periodontal status in CKD patients highlights the interconnection between systemic and oral health. The findings that patients with higher levels of these renal markers exhibited moderate to severe periodontitis suggest that the systemic burden of CKD may exacerbate periodontal disease. The exploration of the association between salivary calbindin-D28k levels and oral health in patients undergoing renal dialysis, along with its relationship to renal and LFTs, reveals important insights into the role of this calcium-binding protein in the context of CKD.

The significant association between salivary calbindin-D28k levels and periodontal scoring in renal dialysis patients suggests a close link between calcium metabolism and periodontal health. Periodontal disease is a common complication in CKD patients, particularly those undergoing dialysis, due to a combination of factors such as immunosuppression, altered calcium-phosphate metabolism, and poor oral hygiene. The significant association observed in Table 7A underscores the potential role of calbindin-D28k in maintaining periodontal health, possibly through its involvement in calcium homeostasis and the maintenance of periodontal tissue integrity. The continuation of the association between salivary calbindin-D28k levels and RFTs further supports the idea that disrupted calcium metabolism in CKD patients has systemic implications, including effects on oral health. The significant result observed between RFT and salivary calbindin-D28k levels ( Table 7B ) indicates that as renal function declines, there may be corresponding changes in the levels of calbindin-D28k in saliva. This could be due to impaired renal synthesis or altered regulation of calcium-binding proteins in response to declining kidney function. The kidneys play a pivotal role in calcium and phosphate balance, and their dysfunction could lead to alterations in the production or secretion of calbindin-D28K, affecting its availability in saliva and thus influencing oral health outcomes. However, the lack of a significant association between LFTs and salivary calbindin-D28k levels ( Table 7B ) suggests that liver function may not directly influence the levels of this protein in saliva in the same way that renal function does. While the liver is involved in various metabolic processes, including the synthesis of certain proteins, the specific regulation of calbindin-D28k appears to be more closely tied to renal function rather than hepatic function in the context of CKD. 37 This finding highlights the importance of focusing on renal-related factors when assessing the role of calbindin-D28k in CKD patients. The differential association of salivary calbindin-D28k with RFT, but not LFT, also points to the possibility that salivary calbindin-D28k could serve as a biomarker for monitoring renal function and its impact on oral health in dialysis patients.

Studying calbindin-D28k in both serum and saliva and correlating various biochemical levels with dental caries prevalence from the time of diagnosis of CKD till the completion of treatment in hospital setup, followed by oral hygiene instructions in these patients, will give us better prospective which is a possible limitation here. The correlation analysis between intracellular calbindin-D28k levels in patients undergoing renal dialysis and their oral health, alongside key biochemical parameters, offers further insights into the complex interactions between systemic health, calcium metabolism, and oral health result in CKD patients. The significant positive correlations observed between intracellular calbindin-D28k levels and biochemical parameters such as sodium and RBS highlight the potential systemic implications of altered calcium-binding protein levels in CKD patients. Sodium levels are critical in maintaining fluid balance and blood pressure, which are often dysregulated in CKD. The positive correlation with calbindin-D28k suggests that this protein may play a role in modulating sodium levels, possibly through its involvement in calcium-dependent cellular processes. Similarly, the correlation with RBS could indicate a relationship between calcium homeostasis and glucose metabolism, both of which are often disrupted in CKD patients, particularly those with concomitant diabetes. Conversely, the significant negative correlation between calcium levels and salivary calbindin-D28k indicates a possible compensatory mechanism where reduced calcium availability in the blood might lead to increased reliance on calcium-binding proteins such as calbindin-D28k. This inverse relationship suggests that as calcium levels decrease, the body might upregulate calbindin-D28k to maintain cellular calcium homeostasis, which could have implications for both systemic and oral health. The continued correlation analysis revealed a significant negative correlation between salivary calbindin-D28k levels and periodontal scoring in CKD patients with poor oral health. This finding indicates that higher levels of salivary calbindin-D28k are associated with better periodontal health, as reflected by lower periodontal scores.

## Conclusion

**Fig. 3:**
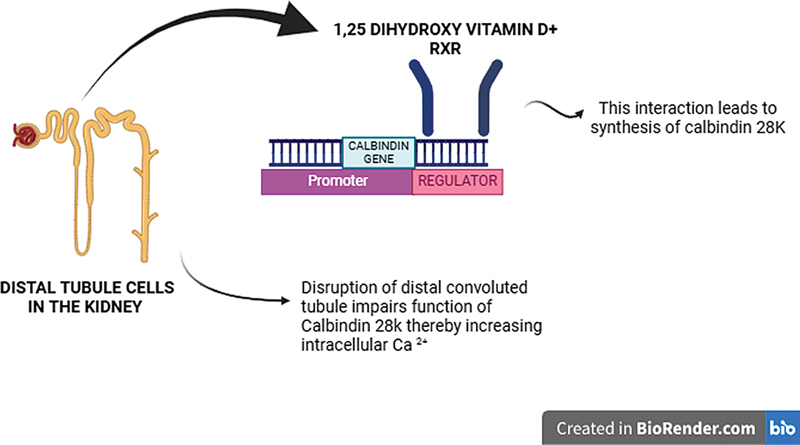
Depicts the effect of chronic kidney disease on distal convoluted tubule and its consequences through calbindin-D28k.

The study confirms that low levels of calbindin-D28k in CKD patients with poor health can be used as a prognostic marker for CKD. Estimating calbindin-D28k levels can help predict future CKD and prevent it in subjects with poor oral health. Impaired function of calbindin-D28k proportionally increased intracellular calcium ions that have detrimental effects. In CKD patients the DCT is effected, therefore renal function of 1,25-dihydroxy vitamin D is also impaired, which is crucial for calbindin-D28k production ( Fig. 3 ).
